# Ion Channels and Ionotropic Receptors in Astrocytes: Physiological Functions and Alterations in Alzheimer’s Disease and Glioblastoma

**DOI:** 10.3390/life13102038

**Published:** 2023-10-11

**Authors:** Annamaria Lia, Alessandro Di Spiezio, Lorenzo Vitalini, Manuela Tore, Giulia Puja, Gabriele Losi

**Affiliations:** 1 Department Biomedical Science, University of Padova, 35131 Padova, Italy; annamaria.lia@unipd.it (A.L.); alessando.dispiezio@unipd.it (A.D.S.); 2 Neuroscience Institute (CNR-IN), Padova Section, 35131 Padova, Italy; 3 Department Life Science, University of Modena and Reggio Emilia, 41125 Modena, Italy; 267888@studenti.unimore.it (L.V.); giulia.puja@unimore.it (G.P.); 4 Institute of Nanoscience (CNR-NANO), Modena Section, 41125 Modena, Italy; manuela.tore@unimore.it; 5 Department Biomedical Science, Metabolic and Neuroscience, University of Modena and Reggio Emilia, 41125 Modena, Italy

**Keywords:** astrocytes, ion channels, glia, Alzheimer’s disease, glioblastoma

## Abstract

The human brain is composed of nearly one hundred billion neurons and an equal number of glial cells, including macroglia, i.e., astrocytes and oligodendrocytes, and microglia, the resident immune cells of the brain. In the last few decades, compelling evidence has revealed that glial cells are far more active and complex than previously thought. In particular, astrocytes, the most abundant glial cell population, not only take part in brain development, metabolism, and defense against pathogens and insults, but they also affect sensory, motor, and cognitive functions by constantly modulating synaptic activity. Not surprisingly, astrocytes are actively involved in neurodegenerative diseases (NDs) and other neurological disorders like brain tumors, in which they rapidly become reactive and mediate neuroinflammation. Reactive astrocytes acquire or lose specific functions that differently modulate disease progression and symptoms, including cognitive impairments. Astrocytes express several types of ion channels, including K^+^, Na^+^, and Ca^2+^ channels, transient receptor potential channels (TRP), aquaporins, mechanoreceptors, and anion channels, whose properties and functions are only partially understood, particularly in small processes that contact synapses. In addition, astrocytes express ionotropic receptors for several neurotransmitters. Here, we provide an extensive and up-to-date review of the roles of ion channels and ionotropic receptors in astrocyte physiology and pathology. As examples of two different brain pathologies, we focus on Alzheimer’s disease (AD), one of the most diffuse neurodegenerative disorders, and glioblastoma (GBM), the most common brain tumor. Understanding how ion channels and ionotropic receptors in astrocytes participate in NDs and tumors is necessary for developing new therapeutic tools for these increasingly common neurological conditions.

## 1. Introduction

Astrocytes constitute the most abundant glial cell population in the brain. Astrocytes are heterogeneous and highly complex cells (Verkhratsky and Nedergaard (2018) [1]; Khakh and Deneen (2019) [2]) and they develop in strict and dynamic interaction with neurons. Indeed, astrocytes govern synaptogenesis and synapse maturation and elimination through different active molecules and phagocytic functions (Khakh and Sofroniew (2015) [3]; Allen and Eroglu (2017) [4]). During development and throughout the whole life span, astrocytes play a plethora of crucial functions that include control of tissue homeostasis, metabolism, and neurovascular coupling (Allaman et al. (2011) [5]; Allen and Lyons (2018) [6]; Lia et al. (2023a) [7]). 

Neuronal, glial, vascular, and extracellular matrix elements contribute to forming the functional unit responsible for information processing and brain functions that was recently named the “brain active milieu” (Semyanov and Verkhratsky (2021) [8]), which is largely affected in brain diseases. Indeed, in neurodegenerative diseases (NDs), gliosis is a common hallmark, with both astrocytes and microglia playing a major role. 

Astrocytes and microglia rapidly transform into a reactive state characterized by morphological, transcriptional, and functional changes that frequently lead to neuroinflammation (Pekny et al. (2016) [9]; Liddelow and Barres (2017) [10]; Sofroniew (2020) [11]; Escartin et al. (2021) [12]). The molecular signature of these dynamic changes is only partially understood. Mixed and even opposing effects may be produced by the same activated glia depending on the specific context and factors that are the focus of several ongoing studies (Escartin et al. (2021) [12]). Reactive astrocytes may release protective cytokines and neurotrophic factors that may reduce cellular damage and, eventually, restore tissue physiological functions, especially after acute events such as stroke or brain trauma. 

In chronic NDs, instead, activated astrocytes and microglia persist in a reactive state that may exert detrimental effects by producing excessive reactive oxygen or nitrogen species (ROS or RNS, respectively), as well as proinflammatory cytokines and neurotoxic factors (Guttenplan et al. (2021) [13]; Brandebura et al. (2023) [14]; Patani et al. (2023) [15]). 

In addition to NDs, astrocytes are involved in glioblastoma (GBM) genesis and progression and in glioblastoma-related symptoms due to the alterations of local glial and neuronal functions (Brandao et al. (2019) [16]; Yang et al. (2022) [17]).

Astrocytic ion channels, in contrast to neuronal ones, were considered important only for the control of ion tissue homeostasis, particularly for potassium (Kuffler et al. (1966) [18]; Orkand et al. (1966) [19]; Kuffler (1967) [20]). Indeed, astrocytes are electrically non-excitable cells since they do not generate action potentials. For this reason, astrocytes were, for a long time, described as passive cells in contrast with neurons, which were considered the only players in synaptic transmission and information processing. This view has changed considerably in the last few decades. In particular, calcium imaging studies revealed that astrocytes are highly active cells capable of releasing gliotransmitters, such as glutamate, ATP, and D-serine. Gliotransmitters dynamically interact with neurons, modulating synaptic transmission, plasticity, and ultimately, behavior (Araque et al. (2014) [21]; Bazargani and Attwell (2016) [22]; Santello et al. (2019) [23]; Lyon and Allen (2021) [24]; Kofuji and Araque (2021) [25]). 

Intracellular calcium transients in astrocytes were initially studied in the soma, where intracellular stores are the main source of these events. Currently, perisynaptic astrocytic processes (PAPs), the ultra-thin processes that intimately contact synapses (Ventura and Harris (1999) [26]; Bushong et al. (2002) [27]), are considered the most active compartment, in which calcium events are far more frequent and complex than in the soma, occurring in localized and heterogeneous events. This calcium activity in small processes relies not only on calcium released from internal stores but also on calcium influx from membrane ion channels (Lia et al. (2021) [28]; Ahmadpour et al. (2021) [29]). 

Finally, the membrane potential was very recently shown to be highly dynamic in PAPs (Armbruster et al. (2022) [30]). These voltage transients may affect, for instance, glutamate uptake and, indirectly, neuronal excitability. Accordingly, although not excitable, astrocytes are now considered electrically active cells (McNeill et al. (2021) [31]). 

**Figure 1:**
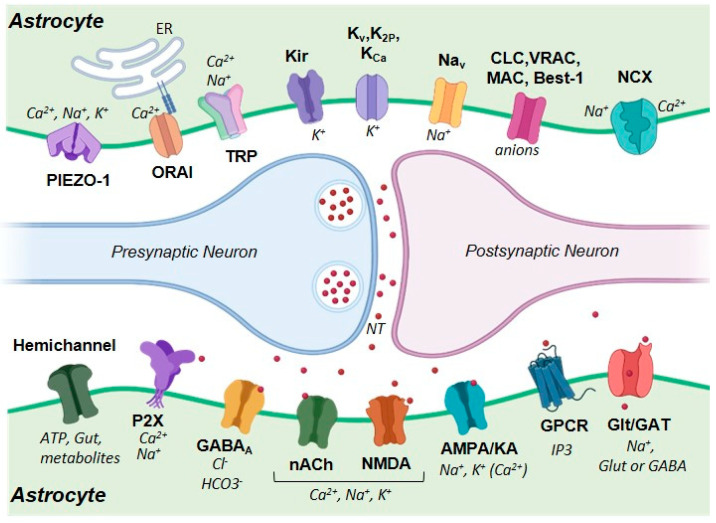
Ion permeable channels and receptors in astrocytes. Schematic view of the main ion channels and ionotropic receptors in astrocyte processes in a tripartite synapse. Ion channels (top), from left to right: tension gated PIEZO-1 channel; endoplasmic reticulum (ER)-STIM1 activated ORAI channels (ORAI-1,-3); TRP channels (TRPV4 is depicted, and others include TRPA1, TRPV1, TRPC1, and TRPC4-6); potassium permeable channels Kir (Kir 4.1, Kir 5.1, and K_ATP_, which include Kir 2.1-3 and Kir 6.1-2), K_v_ (K_v_1.1, K_v_1.6, K_v_3.4, and K_v_4.3), K_2P_ (TREK1-2 and TWIK1), and K_Ca_ (K_Ca_1.1, K_Ca_3.1, and K_Ca_2.1-3, also known as BK, IK, and SK, respectively); sodium channels (Na_v_1.2-3 and Na_v_1.5-6); and anion permeable channels (CLC1-3, VRAC, MAC, and Best-1). The Na^+^Ca^2+^ exchanger (NCX) is also reported. Ionotropic receptors and others (bottom), from left to right: hemichannels composed of Cx 43, 30, and 26; purinegic P2X receptors (P2X_7_); GABA_A_ receptors; cholinergic nicotinic receptors (nACh); and glutamatergic ionotropic receptors (NMDA, AMPA, and KA). Metabotropic receptors coupled to G protein (GPCRs) and electrogenic glutamate and GABA transporters (Glt and GAT1-3, respectively) are also reported. NT, neurotransmitter; Glut, glutamate. Obtained from Biorender.

A summary of ion channels and ionotropic receptors expressed in astrocytes is depicted in Figure 1. Notably, the ion channels and ionotropic receptors expressed in astrocytes and neurons are frequently the same, making the study of their specific roles very difficult. The use of genetically encoded molecules or transgenic animals provides an invaluable solution to this problem.

Ion and water imbalances may affect calcium activity and membrane potential, modulating different intracellular pathways like NF-kB, JAK-STAT3, calcineurin, and NFAT, which are important for astrocytic activation in response to pathological conditions. Furthermore, in NDs, astrocyte modulation of synaptic plasticity is impaired, suggesting a direct involvement of astrocytes in cognitive dysfunctions related to these pathologies (Osborn et al. (2016) [32]). Understanding the functional roles of ion channels and ionotropic receptors in astrocytes, particularly in small processes, may unveil unknown mechanisms of brain function and new therapeutic targets for brain diseases. Indeed, being so deeply affected in NDs, neurons were the focus of the drug research field for many years. Currently, the poor results obtained by neurocentric therapeutic strategies for NDs prompt us to consider glial cells as crucial players and possible new targets. Indeed, astrocytes are largely involved in virtually all brain diseases, including AD, Parkinson’s disease, Huntington’s disease, amyotrophic lateral sclerosis, epilepsy, tumors, ischemia, and injury (Losi et al. (2012) [33]; Pekny et al. (2016) [9]; Brandao et al. (2019) [16]; Khakh and Goldman (2023) [34]; Patani et al. (2023) [15]). 

Here, we will review the most recent advances in this very active field, focusing on astrocytic ion channels in AD (see also Table 1), one of the most diffuse NDs, and in GBM (see also Table 2), a brain tumor that directly involves astrocytes. These two different neurological conditions may serve as examples of how glial cells alterations may contribute to brain pathology. We will first present the role of astrocytic channels in physiological conditions. Then, we will present how their expression and function are modified in AD and GBM. We aim to give an overview of the complex mechanisms underlying two of the most important neurological disorders without forgetting the broader scenario in which many other pathologies show alterations of ion and ionotropic channels in astrocytes.

## 2. Ion Channels and Ionotropic Receptors in Astrocyte Physiology

### 2.1. Potassium Channels

The relevance of K^+^ channels in astrocyte physiology was discovered nearly sixty years ago in seminal studies that revealed a high permeability to this cation at very negative values of resting membrane potential, close to the Nernst’s equilibrium for K^+^ (Kuffler et al. (1966) [18]; Orkand et al. (1966) [19]; Kuffler (1967) [20]; Ransom and Goldring (1973) [113]). Astrocytes express a large set of K^+^-permeable channels, including voltage-dependent channels (Kir), voltage-gated channels (K_v_), voltage-independent two-pore domain channels (K_2P_), and Ca^2+^-dependent channels (K_Ca_) (Seifert et al. (2018) [114]; Verkhratsky and Nedergaard (2018) [1]; McNeill et al. (2021) [31]). Maintenance of K^+^ homeostasis in brain tissue is one of the major known astrocytic functions, and it is mediated by different K^+^ channels together with active Na^+^/K^+^ cotransporters. 

Astrocyte K^+^ channels and transporters mediate the spatial buffering of extracellular K^+^ that is internalized in regions of high neuronal activity and redistributed by gradients in nearby, less active regions through gap junctions that connect astrocytes (Kofuji and Newman (2004) [115]; Verkhratsky and Nedergaard (2018) [1]; McNeill et al. (2021) [31]). Of note, an extracellular K^+^ increase is not due solely to repolarizing axons but also to the activation of postsynaptic N-methyl-D-aspartate (NMDA) receptors, which are K^+^ permeable (Shih et al. (2013) [116]; Tyurikova et al. (2022) [117]), and the activation of γ-aminobutyric acid (GABA) receptors through the involvement of cation chloride cotransporters (Kaila et al. (1997) [118]; Voipio and Kaila (2000) [119]; Viitanen et al. (2010) [120]). Also, for this reason, extracellular K^+^ concentrations are particularly dynamic in active brain regions and need to be properly controlled.

Kir channels are inwardly rectifying channels composed of a family of 16 subtypes, classified into 7 sub-families, expressed by distinct KCNJ genes. The main ion channels expressed by astrocytes are represented by Kir4.1, a weak, inwardly rectifying K^+^ channel expressed exclusively by glial cells (Kofuji and Newman (2004) [115]; Olsen and Sontheimer (2008) [121]; Brasko et al. (2017) [122]). Kir4.1 channels are open at resting membrane potential and contribute to its very negative value. Its voltage dependency is modulated by Mg^2+^ and intracellular polyamines, and it is reduced at depolarized membrane potentials. Kir4.1 is enriched at perisynaptic astrocytic processes and endfeet, i.e., processes contacting blood vessels, and is also present as a heterodimer with Kir5.1 (Hibino et al. (2004, 2010) [123,124]; Tan et al. (2008) [68]). 

Other Kir channels include Kir2.1-3, Kir6.1, and Kir6.2. The latter compose the ATP-sensitive potassium channels (K_ATP_) formed by four pore-forming Kir6.1 and Kir6.2 subunits and four sulfonylurea receptor subunits (Sun and Hu (2010) [125]). These channels participate in the maintenance of extracellular K^+^ homeostasis, allowing the K^+^ entry inside the cells and its release within capillaries or in adjacent regions of low activity. K_ATP_ channels are also reported to exert anti-inflammatory and neuroprotective effects (Hu et al. (2019) [126]; Chen et al. (2021) [127]).

Voltage-activated K^+^ channels include K_v_ 1.1, 1.6, 3.4, and 4.3. Among these delayed-rectifying (KD) channels with higher permeability at depolarized membrane potentials and transient Ka are channels that mediate rapidly activating and inactivating currents (A-type currents).

The family of voltage-independent K_2P_ channels, also known as leak channels, includes 15 different members. Of these, TREK 1, TREK 2, and TWIK 1 channels have been reported in astrocytes. Their role in setting the negative membrane potential is supported by pharmacological inhibition and shRNA approaches (Zhou et al. (2009) [128]; Hwang et al. (2014) [129]). However, experimental evidence is missing in knock-out mouse models (Du et al. (2016) [130]).

Calcium-activated K^+^ channels (K_Ca_), which are also voltage-dependent and named according to their biophysical properties, include big conductance (BK; K_Ca_1.1), intermediate conductance (IK; K_Ca_3.1), and small conductance (SK; K_Ca_2.1-3) channels. Opening of these channels requires both intracellular Ca^2+^ and membrane depolarization. Their localization is mainly reported in perivascular processes and endfeet where they participate in neurovascular coupling (Price et al. (2002) [131]; Filosa et al. (2006) [132]; Carmignoto and Gómez-Gonzalo (2010) [133]; Filosa and Iddings (2013) [134]).

### 2.2. Sodium Channels

Although voltage-dependent Na^+^ channels are typical of excitable cells such as neurons, their expression is also reported in glial cells, including astrocytes. In situ studies revealed the expression of Na^+^ channels such as Nav1.5, the cardiac tetrodotoxin (TTX) resistant channel type, and to a lesser extent Nav1.2, 1.3, and 1.6, with substantial regional differences (Pappalardo et al. (2016) [135]; Verkhratsky and Nedergaard (2018) [1]). The functional roles of these channels are unclear. It is important to remember that these channels modulate the activity of other cotransporters/pumps, such as Na^+^/K^+^ ATPase and glutamate and GABA transporters.

Nav1.5 and Nav1.6 may be important in NDs as their expression is increased in reactive astrocytes (Black et al. (2010) [136]; Zhu et al. (2016) [137]) and microglia (Pappalardo et al. (2016) [135]).

### 2.3. Calcium Permeable Channels

Calcium homeostasis is crucial for many astrocyte functions. Accordingly, astrocyte plasma membrane and intracellular organelles express several calcium channels and calcium-permeable ionotropic receptors. As mentioned above, astrocytes display complex and dynamic intracellular calcium transients that can be evoked by network activity or occur spontaneously. The spatial and temporal pattern of Ca^2+^ transients is very heterogeneous, showing frequent and localized events in small processes and PAPs, which are called Ca^2+^ microdomains (Grosche et al. (1999) [138]), or large events occurring in soma and major processes in response to sustained network activity (Panatier et al. (2011) [139]; Di Castro et al. (2011) [140]; Shigetomi et al. (2013a) [141]; Kanemaru et al. (2014) [142]; Srinivasan et al. (2015) [143]; Bindocci et al. (2017) [144]; Mariotti et al. (2018) [145]; Stobart et al. (2018) [146]; Arizono et al. (2020) [147]) or in pathological conditions (Shigetomi et al. (2019) [148]). Calcium transients may evoke the release of gliotransmitters (such as glutamate, D-serine, and ATP) that finely modulate synaptic functions (Araque et al. (2014) [21]; Bazargani and Attwell (2016) [22] and, ultimately, behavior (Lyon and Allen (2022) [24]; Nagai et al. (2021) [149]; Kofuji and Araque (2021) [25]).

The mechanisms that generate calcium transients are complex and involve both calcium release from intracellular stores and calcium influx from the plasma membrane (Lia et al. (2021) [28]; Ahmadpour et al. (2021) [29]). Intracellular calcium channels and receptors mediate the release of calcium from the endoplasmic reticulum (ER) or other calcium stores such as mitochondria. Calcium release from the ER is triggered by the activation of metabotropic receptors coupled to G protein (GPCRs) and the consequent production of inositol trisphosphate (IP3), which opens IP3 receptors expressed on ER membranes, leading to the final Ca^2+^ release in the cytoplasm. Indeed, astrocytes sense network activity through high-affinity metabotropic receptors of neurotransmitters, including glutamate, GABA, dopamine, and norepinephrine, as well as other messengers such as ATP and purines. Of note, astrocyte GPCRs are associated not only with Gq but also with Gi, as in the case of GABA_B_ receptors, inducing IP3-mediated Ca^2+^ transients (Mariotti et al. (2015, 2018) [145,150]; Durkee et al. (2019) [151]; Caudal et al. (2020) [152]).

Calcium influx from the external space may be mediated by different ion channels, such as transient receptor potential (TRP) channels, Orai channels, mechanoreceptors (Piezo-1), and ionotropic receptors for different neurotransmitters (Lia et al. (2021) [28]; Ahmadpour et al. (2021) [29]). In addition, reverse operation of the Na^+^-Ca^2+^ exchanger (NCX) can also mediate cytosolic Ca^2+^ events (Rose et al. (2020) [153]).

Orai and TRP channels type C (TRPC) take part in store-operated calcium entry (SOCE), a mechanism that involves the concerted action of these plasma membrane ion channels and the ER-Ca^2+^ sensor STIM1 (Figure 1). Under conditions of a reduced calcium content in the ER, STIM1 activates Orai or TRPC channels that allow calcium influx from the extracellular space (Verkhratsky and Parpura (201) [154]; Yoast et al. (2020) [155]). Indeed, astrocytes express functional Orai channels type 1 and 3 (Kwon et al. (2017) [156]) and TRPC receptors. 

TRP channels are a group of ion channels present in different tissues that can be activated either by physical stimuli, such as cell membrane stretch, osmotic pressure, and temperature, or by molecules such as signaling lipids and others found in hot spices. TRP channels expressed by astrocytes include TRPA1, TRPC1, TRPC4-6, TRPV1, and TRPV4, and their functions are only partially understood (Verkhratsky et al. (2014) [157]; Verkhratsky and Nedergaard (2018) [1]; Ahmadpour et al. (2021) [29]). For instance, TRPA1 takes part in calcium activity in fine processes modulating synaptic inhibition in the striatum and long-term potentiation (LTP) in the hippocampus (Shigetomi et al. (2011, 2013b) [158,159]), while TRPV4 channels are involved in the control of local blood flow (Dunn et al. (2013) [160]) and brain ischemia (Sucha et al. (2022) [161]; Tureckova et al. (2023) [162]).

Other calcium-permeable channels are Piezo channels, transmembrane proteins that act as mechanoreceptors. Stretch-induced membrane tension, caused, for instance, by cell swelling, opens Piezo channels, which are permeable to calcium, potassium, and sodium (Lewis and Grandl (2015) [163]). The role of Piezo-1 channels in glial cells is starting to emerge (Benfenati et al. (2011) [164]; Blumenthal et al. (2014) [165]; Chi et al. (2022) [166]).

### 2.4. Anion Channels

Astrocytes express a large number of anion-permeable channels, including voltage-dependent chloride channels (ClC1-3), volume-regulated anion channels (VRAC), Maxi-Cl^-^ channels (MAC), and Ca^2+^-dependent Cl^-^ channels like Bestrophin 1 (Best1), which are all involved in brain diseases (Verkhratsky and Nedergaard (2018) [1]; Elorza-Vidal et al. (2019) [167]).

The most studied voltage-dependent chloride channel in astrocytes is ClC-2 (Makara et al. (2003) [168]), which is widely expressed in all tissues. In astrocytes, the auxiliary subunit GlialCAM modulates ClC-2 electrophysiological properties and subcellular localization (Jeworutzki et al. (2012) [169]). ClC channels are thought to play a role in GABAergic signaling as they favor Cl^-^ exit from astrocytes at rest and especially during swelling (Verkhratsky and Nedergaard (2018) [1]; Elorza-Vidal et al. (2019) [167]).

VRAC channels are formed by leucine-rich repeats containing the 8A subunit (LRRC8A) and at least one other LRRC8 subunit (B-E). VRACs are also known as SWELL1 (Qiu et al. (2014) [170]; Voss et al. (2014) [171]). These channels play a crucial role in cell volume regulation after cell swelling by allowing the efflux of Cl^-^ and other osmolytes, including glutamate and taurine (Mongin (2016) [172]; Osei-Owusu et al. (2018) [173]; Elorza-Vidal et al. (2019) [167]). SWELL1 mediates glutamate release from astrocytes and contributes to the modulation of basal synaptic transmission and excitotoxicity after stroke (Yang et al. (2019) [174]). In addition, astrocytic glutamate release through VRACs evokes slow inward currents in nearby neurons (Gómez-Gonzalo et al. (2018) [175]), which may be involved in pathological conditions, synchronizing the principal cells and favoring seizure onset (Fellin et al. (2004) [176]; Gómez-Gonzalo et al. (2010) [177]). Recently, it was shown that astrocytes in the ventral tegmental area (VTA) release GABA through SWELL1, modulating dopaminergic neuron disinhibition in cocaine-induced rewards (Yang et al. (2023) [178]).

MAC channels are anion channels with very high single-channel conductance that is permeable to anions such as Cl^−^, pyruvate, glutamate, and ATP (Lalo et al. (2014) [179]; Sabirov et al. (2021) [180]). The core of these channels was recently shown to be SLCO2A1, which is also known to be a prostaglandin transporter (Kanai et al. (1995) [181]; Sabirov et al. (2017) [182]). In astrocytes, MAC channels are gated by swelling or hypoxia, releasing ATP (Liu et al. (2006, 2008) [183,184]). Therefore, different pathological conditions can enhance the opening of these channels.

Best1 is a calcium-dependent anion channel largely expressed on astrocytes, particularly on PAPs (Woo et al. (2012) [185]; Park et al. (2013) [186]). Best1 is permeable to chloride and anions like glutamate and GABA. This channel was shown to modulate synaptic plasticity and GABA tonic currents, affecting network activity and also cognitive functions in pathological conditions such as AD (Lee et al. (2010) [187]; Yoon et al. (2011, 2012) [188,189]; Jo et al. (2014) [52]; Park et al. (2015) [190]; Kwak et al. (2020) [191]).

### 2.5. Ligand-Gated Ion Channels (Ionotropic Receptors)

Astrocytes express several ionotropic receptors, i.e., ligand-gated ion channels, whose functions are only partially understood. The main known ionotropic receptors reported in astrocytes are glutamatergic (iGluRs), GABAergic (GABARs), nicotinic cholinergic (nAChRs), purinergic (P2XRs), glycinergic, and serotonergic receptors (Verkhratsky and Nedergaard (2018) [1]).

iGluRs include α-amino-3-hydroxy-5-methyl4-isoxazolepropionic acid (AMPA), Kainate (KA), and NMDA receptors. Astrocytic AMPA receptors are expressed in the cortex, cerebellum, hippocampus, olfactory bulb, and spinal cord, with different GluA1-4 subunit compositions (Höft et al. (2014) [192]; Mölders et al. (2018) [193]). AMPARs in astrocytes have been reported to modulate glutamate transporters (López-Bayghen et al. (2003) [194]) and motor coordination (Saab et al. (2012) [195]). KARs in astrocytes were reported to be involved in a model of temporal lobe epilepsy (Vargas et al. (2013) [196]); however, their physiological role is unknown.

Although initially controversial, likely due to a lower expression compared to other iGluRs, the presence of NMDARs in astrocytes is now established. Functional NMDA receptors are reported in astrocytes of the cortex, spinal cord, hippocampus, and cerebellum (Verkhratsky and Kirchhoff (2007) [197]; Verkhratsky and Nedergaard (2018) [1]). In astrocytes, NMDARs are composed of GluN1, GluN2A-D, and GluN3 subunits, mainly in combinations that limit Mg^2+^ blocks. The role of NMDARs in astrocytes has been poorly explored (Skowrońska et al. (2019) [198]).

Astrocytes express both ionotropic GABA_A_ and metabotropic GABA_B_ receptors. GABA_A_ receptors are pentameric ion channels that bind to γGABA and are permeable to Cl^–^ and HCO_3_^−^ ions. Depending on the intracellular Cl^–^ concentration, activation of GABA_A_Rs mediates either hyperpolarization or depolarization. Indeed, their activation hyperpolarizes mature neurons, whereas it depolarizes glial cells and immature neurons (Labrakakis et al. (1998) [199]). Typically, GABA_A_ receptors are composed of five subunits selected from a pool of 19 isoforms, including two α, two β, and a third type of subunit. The functional and pharmacological characteristics of GABA_A_ receptors in astrocytes, which depend on their subunit composition, are only partially known (Müller et al. (1994) [200]; Fraser et al. (1995) [201]; Höft et al. (2014) 95]). Functionally, astrocytic GABA_A_Rs are thought to modulate extracellular Cl^-^ concentrations, thus indirectly modulating GABAergic signaling (Egawa et al. (2013) [202]), intracellular pH, and K^+^ influx (Ma et al. (2012) [203]). Notably, astrocytes synthesize neurosteroids (Puia and Belelli (2001) [204]; Belelli and Lambert (2005) [205]; Reddy (2010) [206]), potent modulators of GABA receptors with paracrine and possible autocrine effects, although these latter effects are largely unexplored.

Nicotinic acetylcholine receptors are pentameric ligand-gated ion channels composed of different subunits (α2-10 and β2-4) and expressed by neurons and astrocytes in different brain regions (Gotti et al. (2006) [207]; Shen and Yakel (2012) [208]; Zoli et al. (2018) [209]). Neuronal nAChRs are involved in gene expression, neurotransmitter release, and synaptic plasticity, thus affecting learning and memory (Gotti et al. (2006) [207]; Zoli et al. (2018) [209]; Koukouli and Changeux (2020) [210]). Astrocytes express mainly α7s or α4β2 receptors. Activation of α7-nAChRs triggers calcium transients in astrocytes and the release of gliotransmitters such as glutamate, GABA, ATP, and D-serine, which affect neuronal and synaptic activity (Pirttimaki et al. (2013) [56]; Wang et al. (2013) [211]; Papouin et al. (2017) [212]; Lezmy et al. (2021) [213]). Of note, nAChRs are also actively involved in neuroinflammation by acting on NfkB and STAT3 and could represent a target for novel therapeutic strategies (Wang et al. (2003) [214]).

Purinergic receptors are largely expressed in astrocytes and glial cells, where they play a major role in different pathological conditions (Fields and Burnstock (2006) [215]; Burnstock (2018) [216]; Huang et al. (2021b) [217]), being suitable targets for future therapies. Purinergic receptors include G-protein coupled (P2Y) and ionotropic (P2X) receptors, nonselective cation channels with high Ca^2+^ permeability. ATP can bind to P2X_7_R, opening the channel and leading to the influx of Ca^2+^ and Na^+^ and the efflux of K^+^. 

Purinergic signaling is actively involved in brain disease. Indeed, ATP and its breakdown products ADM, AMP, and adenosine are enriched in extracellular space in response to cellular damage and a tumor microenvironment (TME), acting as damage-associated molecular patterns (DAMPs), contributing to inflammatory responses (Di Virgilio et al. (2020) [218]; Huang et al. (2021b) [217]; Engel et al. (2022) [219]).

### 2.6. Other Channels

#### 2.6.1. Aquaporins

Aquaporins (AQPs) are a class of transmembrane proteins forming a pore responsible for the bi-directional passage of water and small solutes across the cell membranes while preserving ion gradients. To date, 13 members of this family have been identified in mammals (AQP0-12) (Ishibashi et al. (2017) [220]). AQP4 is expressed mainly by astrocytes in small processes in the proximity of the subarachnoid area, ventricular spaces, and blood vessels (Nielsen et al. (1997) [221]; Aoyama et al. (2012) [222]). Accumulated evidence suggests that AQP4 is necessary not only for water homeostasis but also to clear small molecules and metabolites, including Aβ and Tau proteins, from interstitial space (Iliff et al. (2012, 2014) [223,224]; Kress et al. (2014) [225]). Indeed, impaired AQP expression/function is involved in different neurological conditions affecting the blood-brain barrier (BBB) or causing water accumulation and brain edema (Vella et al. (2015) [226]; Filippidis et al. (2016) [227]; Clément et al. (2020) [228]).

#### 2.6.2. GAP Junctions and Hemichannels

Astrocytes are known to be interconnected in a network by gap junctions, large pores formed by the juxtaposition of two connexons expressed in adjacent astrocytes, that allow intercellular exchange of ions and molecules, including second messengers and metabolites (Pannasch and Rouach (2013) [229]; Giaume et al. (2013) [230]). Connexons are formed by six subunits named connexins (Cx), such as Cx43, Cx26, and Cx30, whose opening depends on intracellular calcium, pH, phosphorylation, or molecules like spermine and spermidine (Saez et al. (2003) [231]; Harris (2007) [232]; Houades et al. (2008) [233]; Decrock et al. (2015) [234]).

Connexins, as well as pannexins, can also form plasma membrane pores called hemichannels, which are not connected to an adjacent astrocyte. Hemichannels may release molecules such as ATP, glutamate, and metabolites into the extracellular space in response to changes in extracellular or intracellular calcium, pH, phosphorylation state, and pro-inflammatory cytokines (Giaume et al. (2013) [230]).

Connexins and hemichannels are considered possible new therapeutic targets for different brain diseases (Decrock et al. (2015) [234]; Charvériat et al. (2017) [235]; Xing et al. (2019) [111]), particularly for epilepsy (Mylvaganam et al. (2014) [236]; Li et al. (2019) [237]; Guo et al. (2022) [238]) and cancer (Sinyuk et al. (2018) [239]; Zhou et al. (2023) [240]).

## 3. Alzheimer’s Disease

Alzheimer’s disease is a common neurodegenerative disease affecting more than 55 million people worldwide (World Health Organization) with a constantly growing incidence. Although different hypotheses have been proposed to clarify the etiopathology of the disease, a complete picture of the underlying mechanisms is missing, as well as an efficient treatment to block AD progression. 

The hallmark of AD is the presence of aggregated amyloid-β (Aβ) plaques and neurofibrillary tangles of phosphorylated tau (Henstridge et al. (2019) [241]). Reactive astrocytes and neuroinflammation are always present in AD (Chun and Lee (2018) [242]; Perez-Nievas and Serrano-Pozo (2018) [243]; Habib et al. (2020) [244]; Viejo et al. (2022) [245]), leading progressively to significant synapse and neuronal dysfunction and, ultimately, to their loss. Currently, neurotoxicity is thought to be linked to extracellular Aβ_42_ oligomers present since disease onset rather than to the plaques that occur at later stages of the disease. Although the large majority of AD is idiopathic, in a small percentage of cases, AD is due to a hereditary component related to mutations in genes involved in Aβ production. In more detail, familial forms of AD are related to the genes encoding amyloid precursor protein (APP) or presenilin-1 and presenilin-2 (PS1 and PS2, respectively), both implicated in APP cleavage. These mutations are useful for obtaining genetic mouse models of the disease that recapitulate many of its features (Webster et al. (2014) [246]).

The involvement of astrocytes in AD pathogenesis is linked to their importance in oxidative stress and neuroinflammatory responses, as supported by several studies performed both in animal models and in humans (Pekny et al. (2016) [9]; Nanclares et al. (2021) [247]). In humans, higher blood levels of glial fibrillary acidic protein (GFAP), a marker of astrocyte reactivity, have been reported in AD patients compared to controls (Abdelhak et al. (2022) [248]). Moreover, a study revealed a positive correlation between cortical Aβ deposition and GFAP levels in symptomatic AD patients (Asken et al. (2020) [249]).

### 3.1. Potassium Channels

Several studies have shown alterations of K^+^ channels in AD. Kir4.1 KO animal models, despite the exclusive Kir4.1 expression in macroglia, showed neuronal hyperexcitability associated with neurodegenerative diseases, including AD (Nwaobi et al. (2016) [40]). Accordingly, postmortem human AD brains showed reduced Kir4.1 levels (Wilcock et al. (2009) [41]). Comparing multiple AD mouse models, Wilcock and colleagues concluded that reduced levels of Kir4.1 mRNA and protein are associated with cerebral amyloid angiopathy (CAA), a condition that affects 70% of AD patients (Kalaria and Ballard (1999) [250]). Finally, as for AQP4, it was proposed that decreased levels of both AQP4 and Kir4.1 are a consequence of the diminished expression of anchoring protein dystrophin 1 (DP71), a protein highly affected by vascular amyloid deposition (Wilcock et al. (2009) [41]). 

Conversely, Huffels et al. revealed that in the APP/PS1 AD mouse model, dentate gyrus astrocytes in the proximity of Aβ plaques showed increased expression of Kir4.1 but regular function, possibly to compensate for increased extracellular K^+^ levels (Huffels et al. (2022) [42]).

K_ATP_ channels expressed in astrocytes and neurons seem to play a critical role during the development of AD. Griffith and colleagues demonstrated that the Kir6.2 subunit is upregulated in astrocytes in the hippocampus of 3 × Tg-AD mice and the postmortem brains of AD patients. Moreover, increased levels of the pore-forming Kir6.2 were found in the plasma membrane of reactive astrocytes of aged 3 × Tg-AD animals (Griffith et al. (2016) [43]). The altered expression levels on the astrocytes’ plasma membranes may affect the resting potential of the cells, resulting in impaired metabolism and gliotransmission. 

In vivo studies showed that inducing the opening of K_ATP_ with diazoxide in 3 × Tg-AD mice results in reduced levels of cortical and hippocampal Aβ oligomers and hyperphosphorylated tau, and improved cognitive performance (Liu et al. (2010) [44]). On the other hand, delivering the K_ATP_ channel blocker glibenclamide to the APP/PS1 hippocampus increases extracellular Aβ levels (Macauley et al. (2015) [45]).

The voltage-gated potassium K_v_3.4 channel subunits are responsible for fast-inactivating K^+^ currents. K_v_3.4 is upregulated in AD human brains and 6-month-old Tg2576 mice, suggesting a possible role in the development of AD (Angulo et al. (2004) [46]; Pannaccione et al. (2007) [47]; Boscia et al. (2017) [48]). In vitro studies performed in primary rat astrocytes showed that Aβ oligomer treatment induces K_v_3.4 expression. Interestingly, silencing K_v_3.4 subunits by intracerebral infusion of selective siRNA was sufficient to reduce both GFAP and Aβ oligomer levels (Boscia et al. (2017) [48]).

Among the K^+^ channels, the intermediate conductance calcium-activated potassium channel 3.1 (K_Ca_3.1) is involved in the control of membrane potential by controlling the K^+^ efflux in response to the inward flow of Ca^2+^ (Yi et al. (2017) [251]). K_Ca_3.1 was found to be up-regulated in astrocytes in senescence-accelerated mouse prone 8 (SAMP8) mice and AD patients (Yi et al. (2016) [49]). Notably, blockage of K_Ca_3.1 with the selective inhibitor TRAM-34 or by genetic ablation was sufficient to reduce astrogliosis and microglia activation in 7-month-old SAMP8 animals and APP-PS1 mice. Moreover, this neuroinflammation reduction was accompanied by an attenuation of memory deficits (Wei et al. (2016) [50]; Yi et al. (2016) [49]; Yu et al. (2018) [51]). Interestingly, in cultured astrocytes, TRAM-34 reduces the Ca^2+^ elevation induced by Aβ oligomers treatment (Yi et al. (2016) [49]). Indeed, it was reported that K_Ca_3.1 is able to induce ER stress by increasing ER Ca^2+^ overload in an Orai1-dependent manner. Accordingly, K_Ca_3.1 gene deletion or pharmacological inhibition prevents astrocyte activation by reducing Ca^2+^-induced ER stress and activating the neuroprotective AKT/mTOR pathway (Yu et al. (2018) [51]).

### 3.2. Calcium Permeable Channels and Intracellular Calcium Activity

The astrocyte role in AD has been extensively studied in relationship with Ca^2+^ activity, with different outcomes present in different AD models. Initial experiments in APP-PS1 mice showed that amyloidosis is linked to an increased spontaneous somatic astrocyte Ca^2+^ activity (Kuchibhotla et al. (2009) [252]) that is P2Y1R-mediated (Delekate et al. (2014) [253]). On the other hand, a diminished sensory-evoked astrocyte response has been recently reported in a different mouse model that is also based on APP and PS1 mutations (Lines et al. (2022) [254]). A reduced astrocyte Ca^2+^ response to locomotion has also been reported in the neocortex of awake-behaving 15-month-old tg-ArcSwe mice (Åbjørsbråten et al. (2022) [255]). These studies were performed at a single time point in AD mouse models.

Conversely, a recent work performed longitudinally in the PS2APP mouse model of AD found that astrocyte Ca^2+^ activity evolves with disease progression, with a drastic reduction of both spontaneous and evoked activity at the onset of Aβ plaque deposition. The reduction of astrocyte Ca^2+^ activity leads to long-term memory impairments. It is noteworthy that by acting on SOCE through astrocyte-specific STIM1 overexpression, both spontaneous and evoked astrocyte Ca^2+^ activity together with synaptic plasticity were rescued (Lia et al. (2023b) [256]). 

Although these studies point to a heterogeneous astrocyte response to different AD-related mutations, AD progression is certainly accompanied by alterations of astrocyte Ca^2+^ activity. Therefore, it is a potential target for therapeutic intervention.

TRPA1 channels mediate Ca^2+^ influx in astrocytes, contributing to free basal Ca^2+^ levels (Shigetomi et al. (2011, 2013b) [158,159]). In 2016, the first study on a mouse model based on APP and PS1 mutants investigated the role of TRPA1 in AD. TRPA1 is upregulated in the late stage of disease in APP/PS1 mice but not in the early phase. Accordingly, knockout (KO) of TRPA1 in APP/PS1 mice improves spatial learning and memory and decreases Aβ deposition (Lee et al. (2016) [35]). Interestingly, another model of AD based on APP and PS1 mutations (APP/PS1-21) showed that, concomitantly with Aβ production, astrocytes became hyperactive. This Ca^2+^ hyperactivity is mediated by TRPA1 channels and is linked to hippocampal CA1 neuronal hyperexcitability (Bosson et al. (2017) [257]). In line with this, chronic pharmacological inhibition of TRPA1 channels has positive effects on disease outcomes in the same AD model by normalizing spine density and maturation and reducing astrocyte Ca^2+^ hyperactivity (Paumier et al. (2022) [36]).

Piezo channels are mechanosensitive cation-conducting channels activated upon increased membrane tension by unknown chemical ligands. It is conceivable that in the context of AD, where Aβ plaque deposition leads to a change in the mechanical environment, Piezo channel function could be significantly altered. Both hippocampal and cortical astrocytes express Piezo1, which allows an increase of cytosolic Ca^2+^ in the presence of mechanical stimuli or upon Yoda1 perfusion, a Piezo1 agonist. Piezo1 activation elicits ATP release by astrocytes, although the mechanisms involved are still unclear (Chi et al. (2022) (46)). 

The role of Piezo1 in the context of AD has been studied in the 5xFAD mouse model, where Yoda1 administration reduced Aβ accumulation and improved synaptic function together with learning and memory, in contrast to selective Piezo1 KO in microglia that exacerbates AD pathology (Hu et al. (2023) [37]). In the 5xFAD model, the authors clearly showed that the protective effect is mediated by microglia, but it has already been shown that astrocyte Piezo1 is upregulated around Aβ plaques in postmortem AD brains (Satoh et al. (2006) [258]; Velasco-Estevez et al. (2018) [259]). Therefore, the role of astrocyte Piezo1 in other AD mouse models could be of potential interest.

### 3.3. Anion Channels

The Best1 channel is highly expressed at astrocyte microdomains in the hippocampus (Woo et al. (2012) [185]), and its role in AD was studied by Jo and co-workers in 2014. The authors showed in 5xFAD and APP/PS1 models of AD an abnormal GABA release in the dentate gyrus mediated by astrocytic Best1. Of note, the authors showed a redistribution of Best1 in the hippocampus of the AD mice. Although Best1 is expressed similarly in control and AD mice, in the AD scenario it is found mainly at the level of the soma and proximal processes. The excess of released GABA activates both ionotropic (GABA_A_) and metabotropic (GABA_B_) receptors with a consequent presynaptic inhibition that affects neurotransmitter release, LTP, and cognitive functions (Jo et al. (2014) [52]).

### 3.4. Ionotropic Receptors

Calcium permeable α7nAChRs are emerging as key players in AD. Cholinergic signaling is deeply affected in AD, and the few medications currently used, like donepezil, rivastigmine, and galantamine, are all acetylcholinesterase inhibitors. AChRs are expressed by all brain cell populations, and the specific role of astrocyte α7nAChRs is under investigation in several laboratories. In particular, it was shown that α7nAChRs expressing astrocytes are increased in the hippocampus and entorhinal cortex of patients with AD but not in other forms of dementia (Teaktong et al. (2003) [53]; Yu et al. (2005) [54]. Other works revealed that astrocytic α7nAChRs are activated by both physiological and pathological concentrations of Aβ, leading to Ca^2+^ transients that favor or oppose synaptic plasticity, respectively (Wang et al. (2002) [55]); Pirttimaki et al. (2013) [56]; Gulisano et al. (2019) [57]). 

Interestingly Tropea et al. showed that α7nAChRs KO mice develop an AD-like phenotype with aging, showing increased Aβ and phospho-tau and cognitive impairment, suggesting that the lack of a physiological target of Aβ, i.e., α7nAChRs, may lead to a compensatory Aβ overproduction that induces neurotoxicity and AD-like symptoms (Tropea et al. (2021) [58]). The specific role of the lack of astrocytic vs. neuronal α7nAChRs is, however, to be determined. 

P2X_7_R expression is upregulated in microglia, both in AD post-mortem brains and AD mouse models (McLarnon et al. (2006) [59]; Martínez-Frailes et al. (2019) [60]), and in astrocytes from mice overexpressing human tau protein (MAPT P301S)(Jin et al. (2018) [61]) and APP/PS1 mice (Martin et al. (2019) [62]). In a recent study, deleting P2X_7_R in an APP/PS1 model of AD had a protective role, reducing the Aβ plaque load and improving spatial memory. The author related this phenotype to a reduced level of chemokine production (i.e., CCL3). Interestingly, P2X_7_R deletion does not affect cytokines production (IL-1β) or microglia activation (Martin et al. (2019) [62]). The authors proposed a new model where high levels of Aβ peptide induce ATP release from microglia and astrocytes (Sanz et al. (2009) [260]; Orellana Roca et al. (2011) [261]), which activates P2X_7_Rs, which in turn triggers the neurodegenerative process, increasing chemokine production, and favoring the pathogenic recruitment of T cells (Martin et al. (2019) [62]). Further studies are needed to verify if the inhibition of chemokine release is sufficient to prevent AD-related impairments.

### 3.5. Aquaporins

The involvement of AQP4 in the development of AD has been reported in several studies. As mentioned above, aquaporins are important for the cerebrospinal fluid (CSF)–interstitial fluid (ISF) exchange. Indeed, it was shown that AQP4 KO mice have a 55% reduction in I^125^-Aβ_40_ washout after intrastriatal injection (Iliff et al. (2012) [224]). The partial clearance of Aβ40 suggests the existence of other pathways involved in Aβ transport and degradation in the brain (Storck et al. 2016 [262]; Gallwitz et al. 2022 [263]). 

Moreover, APP/PS1 mice lacking AQP4 showed increased Aβ deposition, exacerbating AD-related cognitive deficits (Xu et al. (2015) [38]). These observations obtained in animal models were confirmed in patients with AD, where an impaired glia–lymphatic system and mislocalization of astrocytic AQP4 was found (Reeves et al. (2020) [39]). A couple of studies questioned what could be the reason for the AQP4 mislocalization. Evidence supports the idea that the mislocalization of AQP4 on astrocytes is associated with the downregulation of DP71 dystrophin (Wilcock et al. (2009) [41]). Of note, AQP4 mislocalization on astrocytic endfeet could participate in blood-brain barrier (BBB) dysfunction, which in turn contributes to neurodegeneration (Wilcock et al. (2009) [41]; Zlokovic (2011) [264]).

Studies support the idea that, as for Aβ, tau clearance also occurs through the glymphatic system. Indeed, inhibition of AQP4 leads to impaired CSF–ISF exchange and decreased tau clearance (Harrison et al. (2020) [265]). Similar results were found in AQP4 KO mice, in which a severe neurogenic fibrillary pathology is present (Iliff et al. (2014) [223]).

**Figure 2:**
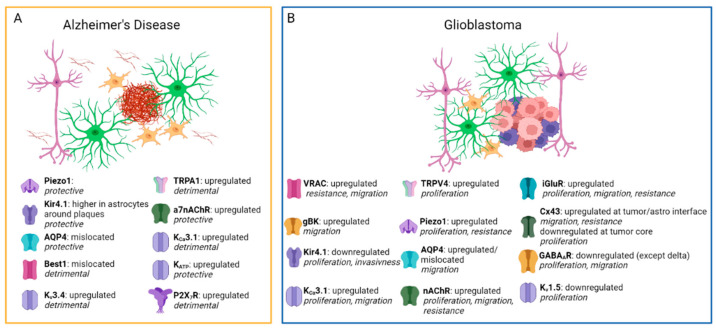
Ion permeable channels and receptors in astrocytes in AD and GBM. (**A**) Schematic view of an Aβ plaque surrounded by reactive astrocytes (green) and microglia (yellow), together with Aβ fibrils and neurons (purple). Ion channels and ionotropic receptors reported in Table 1 are highlighted below with information about their expression changes and the consequent protective or detrimental effects on brain functions. (**B**) Schematic view of GBM cells surrounded by reactive astrocytes (green) and microglia (yellow), together with neurons (purple). The different colors of the GBM cells highlight the GBM cell heterogeneity. Ion channels and ionotropic receptors reported in Table 2 are highlighted below with information about expression changes in GBM cells and their effects in promoting tumor proliferation, migration, or resistance to chemotherapy. Synaptic contacts between neurons are omitted for simplicity. Obtained from Biorender.

In conclusion, ion homeostasis in astrocytes is widely modified in AD (see Table 1 and Figure 2). Notably, the trend shows an increased excitability during the early phases of the disease and a decrease in the late phases, in particular for Ca^2+^ activity. Therefore, trying to revert this trend by targeting ion channels or receptors in astrocytes could help prevent neuronal loss and AD progression.

## 4. Glioblastoma

The astrocytic tumor known as glioblastoma (GBM, grade IV glioma, World Health Organization) is the most aggressive and common glioma in adults (Weller et al. (2015) [266]). GBM has very frequent recurrence and a poor prognosis (14–20 months) due to several factors, including high cellular heterogeneity, invasiveness, microvascular proliferation, therapeutic resistance, and recurrence after surgical removal (Wen et al. (2020) [267]). The existence of a functional network among GBM cells and the tumor microenvironment (TME) preserves and fosters its development (Osswald et al. (2015) [268]; Weil et al. (2017) [269]; Venkataramani et al. (2019) [95]). Indeed, GBM cells are interconnected (between them) through tumor microtubes (TMs) that join single cells via gap junctions (GJs), mainly formed by Cx43, and form an intracellular pathway (Osswald et al. (2015) [268]) with important roles both in tumor progression and in resistance to cytotoxic therapies (Weil et al. (2017) [269]; Li et al. (2020) [270]). 

Furthermore, a tumor–neuron network exists: bona fide synapses were detected between presynaptic neurons and postsynaptic glioma cells, mostly at the TM level, and the crosstalk between neurons and specific tumor cells within the TME is a crucial step in cancer initiation and progression (Jung et al. (2020) [96]). 

The release of glutamate near GM cells from neurons and from neighboring astrocytes, results in complex calcium signaling followed by de novo formation of TMs and increased invasion speed (Venkataramani et al. (2019, 2022 [95,271]). In addition to adjacent neurons and astrocytes, GBM cells themselves secrete glutamate through a cystine/glutamate antiporter (SLC7A11), producing hyper-excitability in the peritumoral zone that fosters GBM malignity (Lo et al. (2008) [272]). 

In support of this hypothesis, it was shown that SLC7A11 upregulation in GBM cells correlates with tumor invasion and a worse outcome in GBM patients, as well as with the onset of tumor-related seizures in patients (Robert et al. (2015) [273]). Moreover, neuronal hyperactivity results in the release of factors, such as brain-derived neurotrophic factor (BDNF) and soluble neuroligin 3 (NLGN3) (Venkatesh et al. (2015) [274]), that add support to glioma progression and facilitate the synapsing of neurons onto glioma cells (Goethe et al. (2023) [275]). 

GBM exhibits intra-tumoral heterogeneity, and a small population of neuronal and neural progenitor-like tumor cells has been identified as responsible for the aggressive behavior of the tumor and its resistance to treatments. Interestingly, these cells resemble migrating neurons during development (Elias et al. (2023) [276]).

Recently, Hausmann et al. showed that one of these subpopulations can generate rhythmic calcium oscillations that are important for driving tumor progression and invasiveness. This small fraction of tumoral cells having “pacemaker activity” express Ca^2+^ activated potassium channels (K_Ca_3.1) that, by triggering calcium signaling, regulate cell proliferation and motility (Hausmann et al. (2023) [71]).

In GBM cells, the altered expression of specific ion channels with consequent ionic misbalance may contribute to tumor growth, progression, and resistance. Indeed, changes in ion channel activity can lead to the dysregulation of the cell cycle and cell growth, inhibition of apoptosis, and enhancement of cell migration and invasion, all of which contribute to malignant transformation (Litan and Langhans (2015) [277]).

Here, we review the current literature on ion channels and ionotropic receptors in GBM cells and nearby interacting populations.

### 4.1. Potassium Channels

These channels have a significant physiological function in GBM cells. In particular, the voltage-dependent large-conductance Ca^2+^-activated BK channels (gBK in glioma) are involved in the aggressive growth and extensive migratory behavior of GBM cells. These channels are overexpressed in malignant gliomas (like GBM), and their expression level is positively correlated with the severity of the tumor (Molenaar (2011) [66]). These channels can contribute to alterations in the shape and volume of glioma cells during their migration into the TME (Wawrzkiewicz-Jałowiecka et al. (2020) [278]). Even though BK channel activation increases the movement of GBM cells by allowing K^+^ efflux, preventing the same conductance from functioning when the cells are at rest does not stop GBM cells from invading. In fact, it was demonstrated that inhibiting BK channels can reduce GBM cell migration only if the intracellular calcium concentration is increased, which results in the BK channels being in the open state (Brandalise et al. (2020) [67]).

The ionic balance of GBM cells is also altered by the downregulation of the constitutively open Kir4.1 channel (Tan et al. (2008) [127]; Brandalise et al. (2020) [67]). The reduction of Kir4.1 expression in GBM leads to a change in cell phenotype, resulting in the increased formation of filopodia that promote cell invasion (Thuringer et al. (2017) [279]). Furthermore, it has been proposed that a small but steady outflow of potassium at the higher resting membrane potential of GBM cells is facilitated by the remaining portion of functional Kir4.1 channels. By doing so, these channels may cooperate with gBK channels in promoting tumor invasion. In fact, recent experiments have shown that when Kir4.1 and BK channels are simultaneously blocked, the migration of GBM cells is reduced (Brandalise et al. (2020) [67]). The depolarization of glioma cells caused by Kir4.1 loss is associated with increased proliferation, whereas introducing Kir4.1 via stable transfection with the resulting repolarization prompts the transition from G2/M to G0/G1 phase, thereby reducing proliferation. It is noteworthy that this reversal effect can be hindered by depolarization with high extracellular K^+^ or by Ba^2+^ usage (Madadi et al. (2021) [69]). Altogether, these findings indicate that Kir4.1 targeting could be a potential treatment for glioblastoma.

In addition, several tumor types, including GBM, exhibit abnormally high levels of K_Ca_3.1, which plays a major role in cellular activation, migration, and proliferation by regulating the membrane potential and Ca^2+^ signaling (Brown et al. (2018) [70]). Specifically, in migrating GBM cells, calcium signaling frequently manifests as oscillations in the intracellular Ca^2+^ concentration that are important for promoting critical processes in the migratory cycle (Brandalise et al. (2020) [67]). Considering this, it has been hypothesized that K_Ca_3.1 channels may contribute to GBM cell migration by generating or modifying the pattern of these calcium oscillations (Catacuzzeno and Franciolini (2018) [72]). Studies have also shown that GBM cells exhibit an increase in the expression of IL-4 and K_Ca_3.1 when exposed to high levels of radiation. This causes the mobilization of calcium within the cells and the transcription of genes that promote invasion (D’Alessandro et al. (2019) [280]). Given the correlation between K_Ca_3.1 overexpression in glioma patients and their poor survival rate (Hausmann et al. (2023) [71]), it is reasonable to consider targeting K_Ca_3.1 as a means of reducing glioma invasiveness and progression. In fact, both in vitro and in vivo experiments have shown that K_Ca_3.1-silencing and using K_Ca_3.1 inhibitors (such as TRAM-34) reduced tumor infiltration, glioma-associated microgliosis and astrogliosis, and increased survival time in mouse glioma models (Brown et al. (2018) [70]).

Moreover, K_v_ subtypes K_v_1.3 and K_v_1.5 have been demonstrated to play a specific role in the growth-related characteristics of normal glial cells, and it has been proposed that glioma subtypes may exhibit varied expression of these channels (Preußat et al. (2003) [73]). Specifically, there is an inverse correlation between the level of expression of K_v_1.5 in astrocytomas and their grade of malignancy, with high expression observed in low-grade astrocytomas and low expression in glioblastoma. Consequently, there is a positive correlation between the high expression of K_v_1.5 and a favorable outcome of patients with GBM. 

No such association was observed for K_v_1.3 expression (Preußat et al. (2003) [73]; Arvind et al. (2012) [74]). However, research has shown that K_v_1.3 inhibition can regulate astrocytes and microglia reactivity in the context of glioma, leading to a decrease in tumor growth with a direct effect on the invasive properties of glioma cells. These results suggest that K_v_1.3 channels could be promising targets for restoring glial cells functioning and reducing the damage caused by glioma to the surrounding brain tissue (Grimaldi et al. 2018). Other studies have demonstrated that K_v_1.3 is expressed in different murine and human glioma cell lines and can be found in both the plasma membrane and mitochondria. In vitro experiments have shown that treatment with K_v_1.3 inhibitors (such as clofazimine, PAPTP, or PCARBTP) can induce cell death in a significant portion of glioma cells. Nonetheless, modifying these drugs and/or the delivery method is necessary to enable the translation of these findings into clinical practice (Venturini et al. (2017) [76]). 

In addition, KAaH1, a homologous Kv1 blocker from scorpion venom, has been discovered to have an impact on K_v_1.3 by preventing the migration and adhesion of U87 cells. As a result, it may serve as a potential therapeutic approach for treating GBM (Aissaoui et al. (2018) [281]).

### 4.2. Calcium Permeable Channels

#### 4.2.1. TRP Channels

Emerging and underexplored targets for GBM therapy are the TRP channels. Changes in the expression of TRP channels have been linked to cancer growth and development. Indeed, they can be useful as diagnostic and/or predictive markers for a variety of tumor types, including glioma (Chinigò et al. (2021) [282]). Changes in calcium homeostasis due to the abnormal function of these channels in tumors are associated with uncontrolled proliferation and resistance to cell death (Huang et al. (2021a) [78]). Among the TRP channels, TRPV4 (transient receptor potential vanilloid 4) expression is considerably higher in malignant glioma compared to both normal brain tissue and low-grade glioma. Furthermore, there is a negative correlation between the expression of TRPV4 and the prognosis of glioma patients (Yang et al. (2020) [77]). All of this suggests that TRPV4 could be an attractive therapeutic target and biomarker for GBM. Notably, a team of researchers investigated the impact of cannabidiol (CBD) on glioma and showed that TRPV4-mediated calcium influx triggered mitophagy, leading to the hypothesis that this might be the primary cause of glioma cell death in response to CBD treatment. Furthermore, the same group provided in vitro and in vivo evidence that a combination of temozolomide (TMZ) and CBD resulted in a powerful antitumoral effect (Huang et al. (2021a) [78]).

#### 4.2.2. Piezo Channels

Other ion channels important in GBM “biology” are the mechanosensitive Piezo ion channels. The stretch-induced membrane tension caused by cell swelling opens the Piezo channels, which then allow the permeation of calcium, potassium, and sodium (Lewis and Grandl (2015) [163]). Piezo1 specifically is overexpressed in aggressive human gliomas, and its expression is inversely correlated with the patient’s prognosis. The mechanical microenvironment provided by ECM stiffening in tumor tissue activates Piezo1, whose activity promotes focal adhesion and activates the integrin/FAK signaling pathway. Piezo1-mediated signaling also regulates cell proliferation and controls the expression of genes involved in ECM remodeling, which further modulates the tissue stiffness. As a result, the harsher environment increases the expression of Piezo1, which enhances the mechanosensory capacity of cancer cells and promotes glioma aggressiveness. These processes create a feed-forward circuit between Piezo1-dependent mechano-transduction and abnormal tissue mechanics in gliomas, exacerbating the disease (Chen et al. (2018) [79]).

### 4.3. Anion Channels

One channel that is highly expressed in GBM cells is the anion channel VRAC, which mediates the flow of chloride triggered by cell swelling. The primary function of VRAC is to restore a normal cell volume, which may be altered due to various pathological conditions. Additionally, VRAC promotes cell shape and volume changes that are required for cell proliferation and migration (Caramia et al. (2019) [63]). In GBM, VRAC arguably plays a major role. This channel can be activated in vivo by severe hypoxia, a condition that occurs in GBM tissues. In this state, following mechanical stress of the plasma membrane, it has been observed that VRAC activation promotes cell migration and resistance to cell death, both of which increase the severity of GBM (Caramia et al. (2019) [63]). Also, another study revealed that lowering the expression of LRRC8A leads to a decrease in the growth of GBM cells and enhances their sensitivity to the chemotherapeutic drugs TMZ and carmustine, which are commonly used in clinical settings (Rubino et al. (2018) [283]). 

Conversely, a different investigation showed that pharmacological VRAC inhibition and LRRC8A knockdown do not have any impact on GBM cell proliferation or migration, suggesting that VRAC may not be necessary for tumor development (Liu and Stauber (2019) [64]). Further investigation is needed to elucidate the roles that this ion channel may play in cell migration and invasion. 

Moreover, VRAC can transport anticancer drugs like cisplatin and carboplatin into the cell, and the selectivity of the substrate, as well as the pharmacology of VRAC, depends on its subunit composition. Notably, one of the subunits of VRAC, the LRRC8D subunit, plays an important pharmacological role, supporting the transport of these anticancer drugs (Planells-Cases et al. (2015) [65]).

### 4.4. Ligand Gated Ion Channels

#### 4.4.1. GABA_A_ Receptors

In GBM cells, all of the different isoforms of GABA_A_ receptor subunits are present and generally down-regulated compared to grade II and III gliomas, except for the up-regulation of the δ subunit (Smits et al. (2012) [83]). GABA_A_R expression in GBM cells is triggered by contacts with neurons (Synowitz et al. (2001) [284]), fostering the hypothesis of communication between the two cell types. Interestingly, in the neurons surrounding the tumor, a loss of expression of the α1 subunit, leading to a reduction of GABAergic input, was reported (Tantillo et al. (2023) [84]). In the peritumoral area, the expression of specific potassium chloride co-transporters (NKCC1 and KCC2) is dysregulated. NKCC1 and KCC2 levels are increased and decreased, respectively, which results in a high intracellular chloride concentration that, when GABA_A_Rs are activated, induces functional excitation (Pallud et al. (2014) [285]; Campbell et al. (2015) [286]). Similarly, GBM cells could be depolarized by high concentrations of GABA present in the TME (Blanchart et al. (2017) [85]), and the increased intracellular calcium may activate several pathways, also involving fibroblast growth factor, which reduces tumor proliferation and growth (Babateen et al. (2015) [287]; Huberfeld and Vecht (2016) [288]; Blanchart et al. (2017) [85]). GABA does not affect the initial phase of tumorigenesis but it limits growth and disease progression (Blanchart et al. (2017) [85]). It has also been shown that GABA activity promotes tumor cell quiescence, which ends when GABA_A_R is blocked. Since GBM produces high levels of GABA, lowering overall GABA levels by tumor resection can possibly result in rapid recurrence by residual GBM cells (Blanchart et al. (2017) [85]). The importance of GABA signaling is supported by the fact that the loss of functional GABA_A_R correlates with the grade of the glioma and the clinical outcome (Smits et al. (2012) [83]).

Since GABA signaling limits tumor growth, it is conceivable that enhancing GABA_A_R function may represent a valuable approach to GBM therapy. Exogenous (benzodiazepines) and endogenous (neurosteroids, NS) substances are able to positively modulate GABA_A_R function (Puia et al. (2012) [289]). It was shown that progesterone and its 5α-reduced metabolite allopregnanolone (Allo) favor the progression of GBM cell lines at nanomolar concentrations (Zamora-Sánchez et al. (2017) [88]) while having opposite effects at high micromolar concentrations (Zamora-Sánchez et al. (2022) [87]; Feng et al. (2022) [89]). Because GBM cells express steroidogenic enzymes and produce NS (Zamora-Sánchez et al. (2017) [88]), it is possible that these endogenous substances, depending on their metabolism, play significant roles in the maintenance and progression of GBM (Pinacho-Garcia et al. (2019) [290]). Indeed, Allo has a therapeutic effect on GBM cell lines, enhancing TMZ inhibition of cell migration and TMZ-induced apoptosis (Feng et al. (2022) [89]). 

On the other hand, we should keep in mind that GABA, by acting as an excitatory neurotransmitter, may bolster the oncogenic effects of the neuronal hyperactivity previously detailed (Venkatesh et al. (2015) [274]). In fact, it has been observed that while GABA appears to inhibit GBM progression, it also stimulates peritumoral neurons to release glutamate, leading to epilepsy (Radin and Tsirka (2020) [86]).

#### 4.4.2. Glutamate Ionotropic Receptors

The GBM microenvironment is characterized by increased glutamatergic signaling, which fuels tumor progression and induces hyperexcitability of the peritumoral neurons. This heightened activity, in turn, may contribute to the genesis of seizures or epilepsy and to widespread neurodegeneration (Venkataramani et al. (2019) [95]; Jung et al. (2020) [96]). GBM cells express iGluRs such as NMDA (Nandakumar et al. (2019) [98]), KAR and AMPAR (in parts, Ca^2+^ permeable AMPAR) (Maas et al. (2001) [291]; Venkataramani et al. (2019) [95]; Venkatesh et al. (2019) [99]). GBM iGluRs may be stimulated either via neuron–tumor synapses or by glutamate released in an autocrine fashion by tumor cells through the cystine–glutamate antiporter (xCT) (Takano et al. (2001) [292]) or by neighboring reactive astrocytes (Sin et al. (2013) [107]). 

The importance of neuronal activity for GBM progression is widely accepted (Corsi et al. (2019) [293]). Specifically, neurons and GBM cells communicate directly through bona fide synapses and also indirectly via paracrine signals such as NLGN3 or BDNF (Venkatesh et al. (2015, 2017) [274,294]). Aside from the direct unidirectional synaptic contacts between neurons and tumor cells, in GBM, there are indirect presynaptic contacts resembling the tripartite synapses between neurons and astrocytes. Glutamatergic signals may induce calcium transients through calcium-permeable AMPARs, activating downstream Akt-PKB pathways that promote GBM growth and invasion (Ishiuchi et al. (2002) [295]; Piao et al. (200) [296]; Venkatesh et al. (2019) [99]). NMDARs are highly expressed in GBM cells, and their activation also contributes to increased intracellular Ca^2+^ levels (Nandakumar et al. (2019) [98]) and the phosphorylation of transcription factors, contributing to GBM survival and migration (Längle et al. 2019 [297]). 

A cross-talk between these calcium-permeable receptors (NMDAR and AMPAR) in the glutamate-rich microenvironment of GBM probably has an important role in tumor progression (Nandakumar et al. (2019) [98]). Both AMPAR and NMDAR activation influence extracellular matrix proteins (Piao et al. (2009) [296]; Ramaswamy et al. (2014) [298]). 

Finally, the involvement of KAR in GBM has been poorly investigated (Lange et al. (2021) [299]). High expression of GluK4 was found in GBM cell lines (Stepulak et al. (2009) [300]), but more studies are needed to shed light on the role of KARs in GBM progression and glioma-associated epilepsy since glutamate released from GBM cells could activate KARs along with AMPARs and NMDARs (Lyons et al. (2007) [97]).

Since GBM, like several cancers of different tissues, relies on glutamate signaling to survive and proliferate (Yu et al. (2017) [301]), pharmacological targeting of iGluRs or the systems responsible for glutamate release may slow the disease progression while simultaneously limiting neurodegeneration and seizure onset. Considering the interdependency between neuronal hyperexcitability and GBM progression, a GBM therapy with iGluRs antagonists acting on both sides (tumor cells and neurons) is particularly suitable. Indeed, an antiepileptic drug with AMPAR antagonistic properties has been successfully employed in clinical trials for GBM in association with radiochemotherapy (Grossman et al. (2009) [100]; Salmaggi et al. (2021) [302]). Because of the biological relevance of calcium-permeable AMPARs in tumor progression, specific inhibitors of these receptors could represent a promising therapeutic strategy for GBM (Venkataramani et al. (2021) [105]). 

NMDAR antagonists, such as memantine, are useful in the management of GBM (Albayrak et al. (2021) [103]), especially considering their neuroprotective activity. For instance, MP1 and MP2 are two NMDA antagonists derived from memantine that increase autophagy in the human U87MG glioblastoma cell line and reduce its proliferative activity (Cacciatore et al. (2017) [101]). Of note, Blyufer et al. highlighted the potential of riluzole, a drug that inhibits glutamate release, as a potential therapeutic tool for GBM (Yamada et al. (2020) [102]; Blyufer et al. (2021) [104]).

#### 4.4.3. Nicotinic ACh Receptors

GBM cells are capable of synthesizing and releasing Ach, which, acting in an autocrine or paracrine fashion, affects proliferation, survival, and tumor invasion (Thompson and Sontheimer (2019) [90]). The expression of most nAchR subunits in GBM is very low, except for the muscle-type α1 and β1 subunits and the neuronal α7 and α1 subunits (Thompson and Sontheimer (2019) [90]; Pucci et al. (2021, 2022) [91,92]). 

AChR activation increases cell invasion by enhancing the activity of matrix metalloproteinase-9 (MMP-9) through a Ca^2+^-dependent mechanism (Thompson and Sontheimer (2019) [90]). A recent study (Pucci et al. (2021) [92]) found that α9 and α5 subunit mRNAs are highly expressed in GBM and that chronic treatment with selective agonists increases the proliferation of tumor cell lines (U87MG and GBM5). By silencing the expression of the α7 and α9 subunits or by applying selective α7 and α9 AChR antagonists, this effect was prevented, thus indicating that the presence of both subunits was required (Pucci et al. (2021, 2022) [91,92]). Stimulation of α9 and α7 nAChRs can activate various intracellular signaling pathways and regulate gene expression through non-conventional metabotropic channel signaling. In GBM cell lines, the activation of α7nAChRs and α9nAChRs has been shown to inhibit cell apoptosis via the EGFR/Akt pathway and the promotion of cell proliferation via the EGFR/ERK pathway (Khalil et al. (2013) [303]; Pucci et al. 2022 (2022) [91]). 

Considering the importance of nAChRs in GBM progression in the last few years, non-selective and selective antagonists of nAchR have been developed and tested on GBM cell lines. For example, Atracurium Besylate (an nAChR antagonist) (Spina et al. (2016) [93]) and StN2/4/8 (α7 and α9/α10 nAChR antagonists) (Pucci et al. (2022) [91]; Bavo et al. (2023) [94]) have been shown to have significant inhibitory effects on GBM cell line vitality. 

### 4.5. Other Channels

#### 4.5.1. Aquaporins

AQPs play a role in enhancing tumor mobility through several mechanisms: cell volume regulation, cell-cell and cell-matrix adhesion, actin cytoskeleton interaction, control of enzymes and molecules involved in extracellular matrix degradation, and ion channel and transporter co-localization. In the case of GBM, the overexpression of AQP-1, -4, and -9 is consistent with their suggested functions in facilitating the mobility, growth, and survival of glioma cells (Varricchio et al. (2021) [80]). AQP4, the primary aquaporin expressed in the CNS, plays a crucial role in maintaining water and potassium homeostasis. In glioblastomas and other types of astrocytomas, AQP4 expression is elevated and redistributed. In fact, in high-grade brain tumors, AQP4 shows a loss of its polarized distribution at the tips of astroglial pedicels, which can contribute to increased tumor cell migration (Vandebroek and Yasui (2020) [81]). In this regard, a research group suggested that high concentrations of T3 thyroid hormone could be effective in decreasing AQP4 in GBM tumor cells, leading to improved outcomes in terms of reducing the migration and growth of brain tumor cells (Costa et al. (2019) [82]).

#### 4.5.2. Connexin 43

Connexin 43 is an important building block of GJs and hemichannels, and its expression, which is very high in astrocytes, is reduced when they undergo malignant transformation (Caltabiano et al. (2010) [106]). Indeed, the amount of Cx43 protein inversely correlates with the level of malignancy of astrocytomas, and its presence is low in GBM (Sin et al. (2016) [107]; Gielen et al. (2013) [112]). Hence, this relationship between Cx43 expression and glioma severity suggests that Cx43 should have a tumor suppressor ability, restraining the proliferation of glioma cells. 

On the other hand, several pieces of evidence have pointed out the capability of Cx43 to enhance the mobility and invasiveness of GBM cells, facilitating their migration from the tumor core into the surrounding tissues (Sin et al. (2016) [107]; Dong et al. (2017) [110]). The ambivalence of Cx43 effects, ranging from tumor suppressor to cell migration booster, can be partly explained by the heterogeneous expression of Cx43 in the GBM mass. In fact, the cells in the core with low Cx43 expression proliferate, while those with high Cx43 levels are expected to migrate (Sin et al. (2016) [107]; McCutcheon and Spray (2022) [109]). The presence of GJs between GBM cells might inhibit migration, whereas GJs between GBM cells and astrocytes, or between astrocytes themselves, promote tumor progression. Indeed, Cx43 expression is enhanced significantly in the glioma-associated astrocytes of the peritumoral zone that have a decisive role in granting the dissemination of tumoral cells (Sin et al. (2016) [107]). Cx43 can foster invasiveness by activating different pathways (i.e., by interacting with different cytoskeletal proteins) and also by promoting the transfer of oncogenic signaling molecules from tumor cells to neighboring astrocytes or among astrocytes (Sin et al. (2016) [107]; Uzu et al. (2018) [108]; McCutcheon and Spray (2022) [109]).

Cx43 is also involved in tumor progression because it increases GBM resistance to apoptosis and has an impact on cell homeostasis through paracrine hemichannels activity (Sin et al. (2016) [107]).

Moreover, Cx43 and GJ/hemichannels may contribute to glioma-associated epileptic activity in the peritumoral zone induced by changes in the tumor microenvironment. In particular, Cx43 may have a role in the regulation of neurotransmitters such as glutamate and ATP (Dong et al. (2017) [110]).

Furthermore, the expression of Cx43 in GBM cells is associated with the development of resistance to TMZ treatment (Gielen et al. (2013) [112]). It has been shown that Cx43-dependent resistance involves both GJ-dependent and GJ-independent mechanisms that influence the invasion and migration of tumor cells. In response to TMZ treatment, Cx43 modulates mitochondrial apoptotic pathways by regulating Bax and Bcl-2 levels and by influencing the release of cytochrome C from mitochondria (Gielen et al. (2013) [112]; Dong et al. (2017) [110]; Xing et al. 2019 [111]).

Considering the different roles played by Cx43 in GBM progression, it will be extremely important in the development of novel therapeutic strategies for GBM treatment (Xing et al. 2019 [111]) to selectively target Cx43 of specific cell types (tumoral or non tumoral), carefully considering the stage of glioma genesis.

In conclusion, the published research in the field converges toward a complex view of “GBM biology.” The influence of ion channels and ionotropic receptors on tumor development, invasiveness, and resistance to therapies is widely recognized, but on the other hand, is extremely complex. The channels/receptors that are differently expressed in GBM and the results of these changes are a composite (see Table 2 and Figure 2) because they affect not only the single tumoral cell but also the multicellular network of the TM that drives tumor growth and resistance to therapy. Since a major reason for the incurability of gliomas by local treatment is the diffuse infiltration of the brain, determined also by changes in channels/receptors, they are important pharmacological targets for new GBM drugs and as powerful prognostic biomarkers. The development of new therapeutic approaches that target ion channels should always consider not only the effect on single cellular entities but also on the whole “GBM network”.

## 5. Conclusions and Perspectives

When considering the poor results obtained by neurocentric drug research for different NDs such as AD, fifteen years ago, the great scientist Ben Barres made the following statement: “Quite possibly saving astrocytes from dying in neurological disease would be a far more effective strategy than trying to save neurons, glia already know how to save neurons, whereas neuroscientists still have no clue” (Barres (2008) [304]). Scientific advancements in the last fifteen years confirmed this assertion. Indeed, it is now clear that astrocytes contribute to brain disorders with several intermingled mechanisms that depend on different factors such as subcellular localization, disease stage, comorbidity, and others. Technical advances over the last years helped to reveal the morphological, functional, and molecular complexity of glial cells (Endo et al. (2022) [305]) and their plethora of functions, including modulation of behavior and cognition in health and disease. Multiphoton or supersolution microscopy, genetic tools, and transcriptomics allow for a deeper understanding of the molecular fingerprint of reactive astrocytes in brain diseases. In addition, astrocyte small processes, which are in strict contact with synapses, have emerged as the most interesting and yet elusive cellular district. 

Finally, human astrocytes are even more complex and heterogeneous than in rodents (Oberheim et al. (2009) [306]; Vasile et al. (2017) [307]), suggesting their great relevance in human brain physiology and pathology. Accordingly, expanding our knowledge on astrocyte mechanisms at different stages of brain disorders is a required step to design new preventive or therapeutic treatments oreand finally prove that acting on glia is an efficient strategy to save brain functions.

## Figures and Tables

**Table 1:** Summary of the main findings reported on ion channels and ionotropic receptors in astrocytes from AD brains.

	Alzheimer’s Disease	
Channel or Receptor	Main Findings	References
TRPA1	Upregulated in the late AD phase.	Lee et al. (2016) [35]
	Pharmacological inhibition or genetic KO ameliorates AD outcomes in mice.	Lee et al. (2016) [35], Paumier et al. (2022) [36]
Piezo1	Pharmacological activation reduces Aβ accumulation and improves plasticity and memory.	Hu et al. (2023) [37]
	Selective KO in microglia exacerbates AD pathology.	
AQP4	AQP4-KO in AD mice increases AB deposition and cognitive deficits.	Xu et al. (2015) [38]
	AQP4 mislocalization has been found in AD patients.	Reeves et al. (2020) [39]
Kir4.1	Kir4.1 KO mice show neuronal hyperexcitability associated with AD.	Nwaobi et al. (2016) [40]
	Kir4.1 is reduced in postmortem AD brains.	Wilcock et al. (2009) [41]
	Dentate gyrus astrocytes around Aβ plaque show higher Kir4.1	Huffels et al. (2022) [42]
K_ATP_	Kir6.2 subunit is upregulated in AD mice and postmortem AD brains.	Griffith et al. (2016) [43]
	Pharmacological activation of K_ATP_ reduces AD hallmarks and cognitive deficits in AD mice.	Liu et al. (2010) [44]
	Pharmacological inhibition of K_ATP_ increases Aβ deposition in mice.	Macauley et al. (2015) [45]
K_v_3.4	K_v_3.4 is upregulated in AD human and mouse brains.	Angulo et al. (2004) [46], Pannaccione et al. (2007) [47], Boscia et al. (2017) [48]
	K_v_3.4 silencing reduces GFAP expression and Aβ loading in mice.	Boscia et al. (2017) [48]
K_Ca_3.1	K_Ca_3.1 is upregulated in AD patients.	Yi et al. (2016) [49]
	K_Ca_3.1 blockade attenuates neuroinflammation and ameliorates cognitive deficits in AD mice.	Wei et al. (2016) [50], Yi et al. (2016) [49], Yu et al. (2018) [51]
Best1	Mediates abnormal GABA release in AD mice hippocampus and affects synaptic plasticity.	Jo et al. (2014) [52]
	Altered localization in astrocytes from AD mice.	
α7nAChR	Higher expression in astrocytes from AD patients.	Teaktong et al. (2003) [53], Yu et al. (2005) [54]
	Activated by Aβ at physiological or pathological concentrations, affecting synaptic plasticity.	Wang et al. (2002) [55], Pirttimaki et al. (2013) [56], Gulisano et al. (2019) [57]
	α7nAChRsKO mice develop an AD-like pathology	Tropea et al. (2021) [58]
P2X_7_R	Upregulated in microglia from both AD mice and postmortem AD brains.	McLarnon et al. (2006) [59]; Martínez-Frailes et al. (2019) [60]
	Upregulated in astrocytes from AD mice.	Jin et al. (2018) [61], Martin et al. (2019) [62]
	P2X_7_R KO in AD mice reduces cognitive deficits and Aβ plaques without affecting microglia.	Martin et al. (2019) [62]

**Table 2:** Summary of the main findings reported on ion channels and ionotropic receptors in GBM.

	Glioblastoma	
Channel or Receptor	Main Findings	References
VRAC	Highly expressed in GBM cells.	Caramia et al. (2019) [63]
	Promotes migration and resistance to apoptosis but is not necessary for tumor development.	Caramia et al. (2019) [63]; Liu and Stauber (2019) [64]
	May transport anticancer drugs like cisplatin and carboplatin.	Planells-Cases et al. (2015) [65]
gBK	Overexpressed in GBM and contributes to aggressive tumor growth and migration.	Molenaar (2011) [66]
	Inhibition reduces tumor migration only when GBM cells are in their active state.	Brandalise et al. (2020) [67]
Kir4.1	Downregulated in GBM cells.	Tan et al. (2008) [68]; Brandalise et al. (2020) [67]
	The remaining portion of functional Kir4.1 channels may cooperate with gBK channels to promote tumor invasion.	Brandalise et al. (2020) [67]
	Kir4.1 loss depolarizes GBM cells and increases tumor proliferation, while Kir4.1 reintroduction reduces proliferation.	Madadi et al. (2021) [69]
K_Ca_3.1	Elevated levels in GBM cells correlate with poor survival.	Brown et al. (2018) [70]; Hausmann et al. (2023) [71]
	Contribute to GBM cell migration by influencing Ca^2+^ signaling.	Catacuzzeno and Franciolini (2018) [72]
	Silencing and inhibition reduce tumor infiltration and improve survival in mouse models.	Brown et al. (2018) [70]
K_v_1.3, K_v_1.5	Inverse correlation between K_v_1.5 expression and glioma malignancy (no such association was observed for K_v_1.3).	Preußat et al. (2003) [73]; Arvind et al. (2012) [74]
	K_v_1.3 inhibition in glial populations regulates astrocyte and microglia reactivity, reducing tumor growth and invasiveness.	Grimaldi et al. (2018) [75]
	K_v_1.3 inhibitors can induce cell death in glioma cells.	Venturini et al. (2017) [76]
TRPV4	Overexpressed in malignant gliomas like GBM. Negatively correlated with the prognosis.	Yang et al. (2020) [77]
	CBD treatment causes TRPV4-mediated calcium influx triggered mitophagy and consequent glioma cell death.	Huang et al. (2021) [78]
PIEZO1	Overexpressed in malignant gliomas like GBM. Negatively correlates with the prognosis.	Chen et al. (2018) [79]
	ECM stiffness activates PIEZO1 and increases its expression, promoting GBM invasiveness.	
AQPs	AQPs facilitate tumor mobility, survival, and growth through diverse mechanisms.	Varricchio et al. (2021) [80]
	AQP4 overexpression and redistribution in GBM are linked to increased cell migration.	Vandebroek and Yasui (2020) [81]
	T3 hormone may decrease AQP4 in GBM cells, leading to reduced tumor growth and migration.	Costa et al. (2019) [82]
GABA_A_R	The majority of GABA_A_R subunits are downregulated in GBM, except for the δ subunit, which is upregulated.	Smits et al. (2012) [83]; Tantillo et al. (2023) [84]
	GABA signaling decreases GBM proliferation and growth, but it may lead to epilepsy development.	Blanchart et al. (2017) [85]; Radin and Tsirka (2020) [86]; Tantillo et al. (2023) [84]
	GABA_A_R activity supports tumor cell quiescence. After tumor resection, its reduced activity may be responsible for GBM recurrence.	Smits et al. (2012) [83]; Blanchart et al. (2017) [85]
	Neurosteroids could modulate GBM cell line biology.	Zamora-Sánchez et al. (2017, 2022) [87,88]; Feng YH. et al. (2022) [89]
nAchR	Few subtypes of nAchR are expressed in GBM cells.	Thompson et al. (2019) [90]; Pucci et al. (2022) [91]
	nAchR controls the survival, proliferation, and infiltrative capacity of GBM.	Thompson et al. (2019) [90]; Pucci et al. (2021) [92]
	Activation of nAchR produces an increase in [Ca^2+^]_int_ that leads to intracellular pathways activation (Akt, ERK).	Pucci et al. (2022) [91]
	nAchR antagonists decrease the viability, proliferation, and migration of GBM cells.	Spina et al. (2016) [93]; Pucci et al. (2022) [91]; Bavo et al. (2023) [94]
iGluRs	Stimulation of iGluRs leads to GBM progression and contributes to seizures in the peritumoral area.	Venkataramani et al. (2019) [95]; Jung et al. (2020) [96]
	NMDAR, AMPAR, and KAR expressed in GBM can be activated by glutamate released by neurons and by GBM itself.	Lyons et al. (2007) [97]; Nandakumar et al. (2019) [98]; Venkataramani et al. (2019) [95]; Venkatesh et al. (2019) [99]
	Ca^2+^ permeable AMPARs and NMDARs promote tumor progression in the glutamate-rich environment of GBM.	Nandakumar et al. (2019) [98]
	iGluR antagonists may slow GBM progression while limiting neurodegeneration and seizure onset.	Grossman et al. (2009) [100]; Cacciatore et al. (2017) [101]; Yamada et al. (2020) [102]; Albayrak et al. (2021) [103]; Blyufer et al. (2021) [104]; Venkataramani et al. (2021) [105]
Cx43	Cx43 expression is reduced in the tumor core and increased at the tumor–astrocyte interface. Due to its heterogeneous distribution, Cx43 acts both as a tumor suppressor and a promoter of cell migration.	Caltabiano et al. (2010) [106]; Sin et al. (2016) [107]; Uzu et al. (2018) [108]; McCutcheon and Spray (2022) [109]
	Cx43 regulates apoptosis, impacts cell homeostasis, and promotes epileptic activity in the peritumoral zone.	Sin et al. (2016) [107]; Dong et al. (2017) [110]; Xing et al. (2019) [111]
	Cx43 expression in GBM cells is associated with the development of resistance to TMZ treatment.	Gielen et al. (2013) [112]

## Data Availability

Not applicable.
